# Primary cutaneous anaplastic large-cell lymphoma resembling infratemporal space infection: a case report

**DOI:** 10.1186/s12903-024-04178-w

**Published:** 2024-04-18

**Authors:** Xiaohan Yan, Wenhao Ren, Shaoming Li, Zhuang Zhu, Ling Gao, Keqian Zhi

**Affiliations:** 1 Department of Oral and Maxillofacial Reconstruction, the Affiliated Hospital of Qingdao University, Qingdao, 266555 China; 2 School of Stomatology, Qingdao University, Qingdao, 266003 China; 3 Key Lab of Oral Clinical Medicine, the Affiliated Hospital of Qingdao University, Qingdao, 266555 China; 4 Department of Oral and Maxillofacial Surgery, the Affiliated Hospital of Qingdao University, Qingdao, 266555 China

**Keywords:** Primary cutaneous anaplastic large-cell lymphoma, Space infection, Case report, Diagnosis, Treatment

## Abstract

**Background:**

Primary cutaneous anaplastic large-cell lymphoma (PC-ALCL) is a rare T-cell lymphoma belonging to the CD30 + T-cell lymphoproliferative disorders. The case of PC-ALCL in the temporal region is exceedingly rare. Herein, we report a case of PC-ALCL involving the temporal region mimicking infratemporal space infection.

**Case presentation:**

A 78-year-old woman presented to maxillofacial surgery service with a 6-month history of swelling and pain in the left side of her face. Laboratory investigations found an elevated C-reactive protein (CRP). Imaging findings showed enlarged lymph nodes and extensive thickening of subcutaneous tissue of the left temples. Based on these findings, the infratemporal space infection was suspected initially. The patient underwent incision and drainage, and we unexpectedly found no pus in the lesion area. Incisional biopsy showed necrosis and extensive involvement of the left temples by a diffuse infiltrate containing large, atypical cells. The tumor cells were positive for CD30, CD3, Ki67. They were negative for ALK (SP8), CD5, CD8, CD20 and PAX5. After considering these findings, a diagnosis of PC-ALCL was rendered. The patient was admitted to the lymphoma department for systemic chemotherapy and no relapse occurred during a follow-up period of six months.

**Conclusions:**

This report suggests that if there are suspicious intraoperative manifestations, carrying out a biopsy simultaneously, using Hematoxylin and eosin (HE) staining, and a comprehensive Immunohistochemistry (IHC) panel are essential to diagnosing PC-ALCL to prevent misdiagnosis.

## Background

Anaplastic large cell lymphoma (ALCL) is a rare type of non-Hodgkin’s lymphoma (NHL), as well as one of the subtypes of T cell lymphoma with ample cytoplasm and pleomorphic nuclei, expressing near-universal levels of the CD30 [1]. According to the 2016 WHO classification, there are four types of ALCL: systemic ALK-positive ALCL (ALK + ALCL), systemic ALK-negative ALCL (ALK − ALCL), primary cutaneous ALCL (PC-ALCL), and breast implant-associated ALCL (BI-ALCL) [2].

Early diagnosis of PC-ALCL is crucial because the clinical stage is directly related to the prognosis. Patients with PC-ALCL generally have a good prognosis. The 5-year survival rate of PC-ALCL is 80–90%, while age over 60 are unfavorable factors. Multifocal disease is more aggressive than localized disease and tends to relapse after systemic chemotherapy [3]. Clinically, most lesions of PC-ALCL manifest on the extremities, followed by the head and neck, and may manifest as nodules, papules, ulcers, and swelling [4, 5], thus differential diagnosis with carcinoma of the facial skin (e.g. squamous cell carcinoma (SCC) and basal cell carcinoma(BCC)), pyogenic granuloma, and space infection should be considered [6, 7]. Although these diseases might have similar clinical manifestations, they have distinct histopathological features that can be distinguished through pathological examination such as Hematoxylin and eosin (HE) staining, immunohistochemistry (IHC) analysis and cytological examination [8]. Depending on the specific type of disease, various treatment modalities such as surgery, radiotherapy, chemotherapy, curettage and laser are used [9]. The diagnosis of PC-ALCL is based on histopathological and IHC evaluation. It is characterized by ample cytoplasm and pleomorphic nuclei, expressing near-universal levels of the CD30 [10]. Most cases of PC-ALCL present as isolated lesions that can be effectively treated with radical surgical excision or radiation. Multi-agent systemic chemotherapy is typically reserved for cases with increased nodal involvement or a greater extent of widespread disease [11].

Here, we present a case of ALCL in a 78-year-old woman with temple and temporal region involvement, first thought to be an infratemporal space infection. Therefore, we summarized medical history, specialty examination, clinical manifestations and treatment, hoping to provide essential clues for diagnosing PC-ALCL with first symptoms in rare sites.

## Case presentation

A 78-year-old woman presented to maxillofacial surgery service with a 6-month history of swelling and pain in the left side of her face, which got worse in the past 20 days. 6 months ago, a biopsy was performed at another hospital, and the diagnosis was a chronic inflammatory. The patient had a 5-year history of diabetes mellitus.

Clinical examination revealed diffused left-sided temporal swelling and unobvious left parotid swelling, both with tough texture, obvious tenderness, and poor activity.

Laboratory investigations found an elevated C-reactive protein (CRP) concentration of 109 0.97 mg/l and a mildly raised procalcitonin concentration of 0.08 ng/ml.

**Fig. 1:**
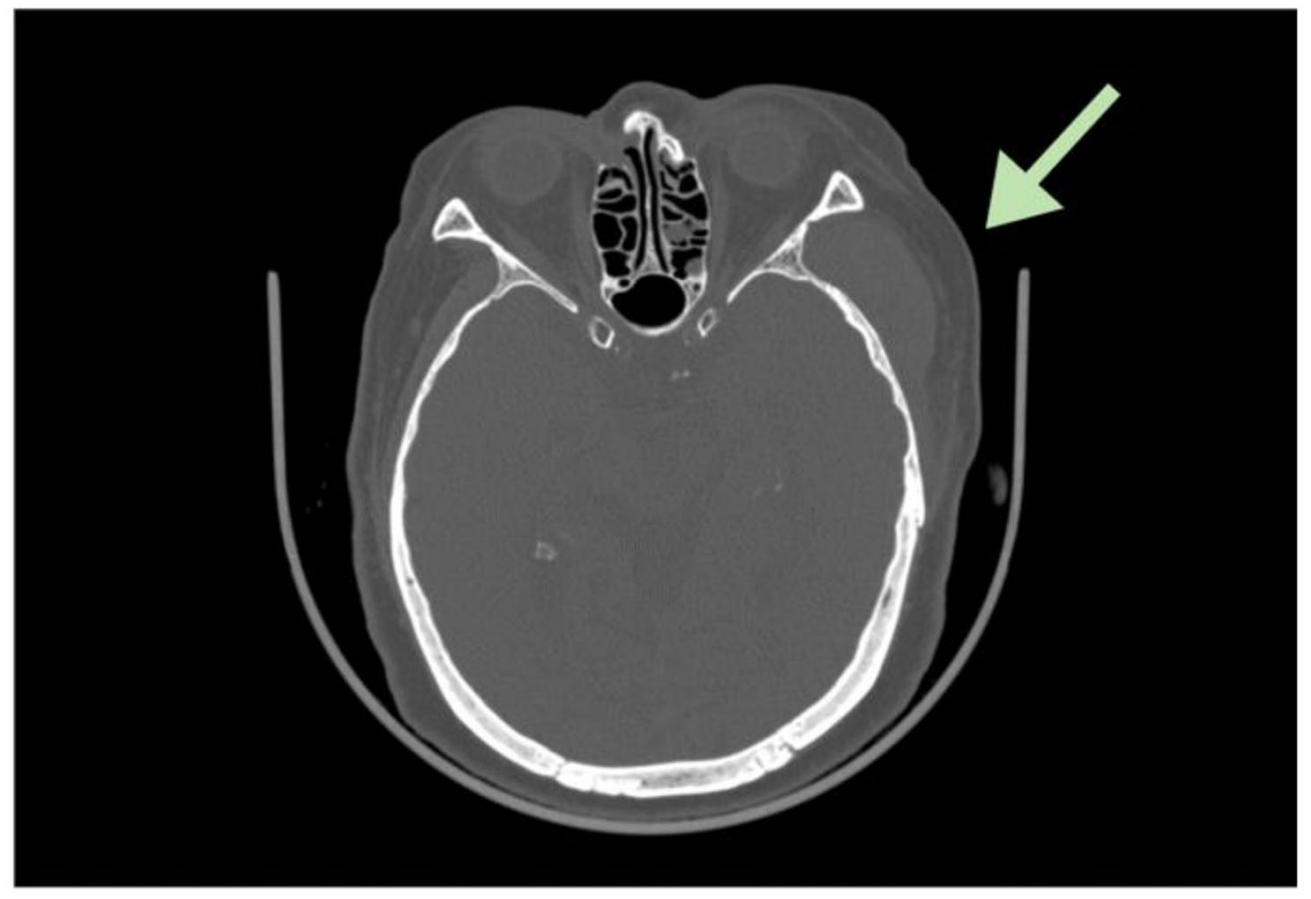
Axial CT scan shows an extensive thickening in the subcutaneous tissue of the left temples

A cone beam computed tomography (CBCT) showed enlarged lymph nodes of levels I to II in the left neck, extensive thickening of subcutaneous tissue of the left temples [Fig. 1] and a parotid hypodense lesion together with a blurred image of masticator, buccal, and submandibular space. Considering these findings, we suspected an infratemporal space infection. Based on these, an incision and drainage were planned for the patient. However, we found the lesion area with only a small amount of clear fluid rather than pus. 4 days later, the pain reappeared in the lesion area.

**Fig. 2:**
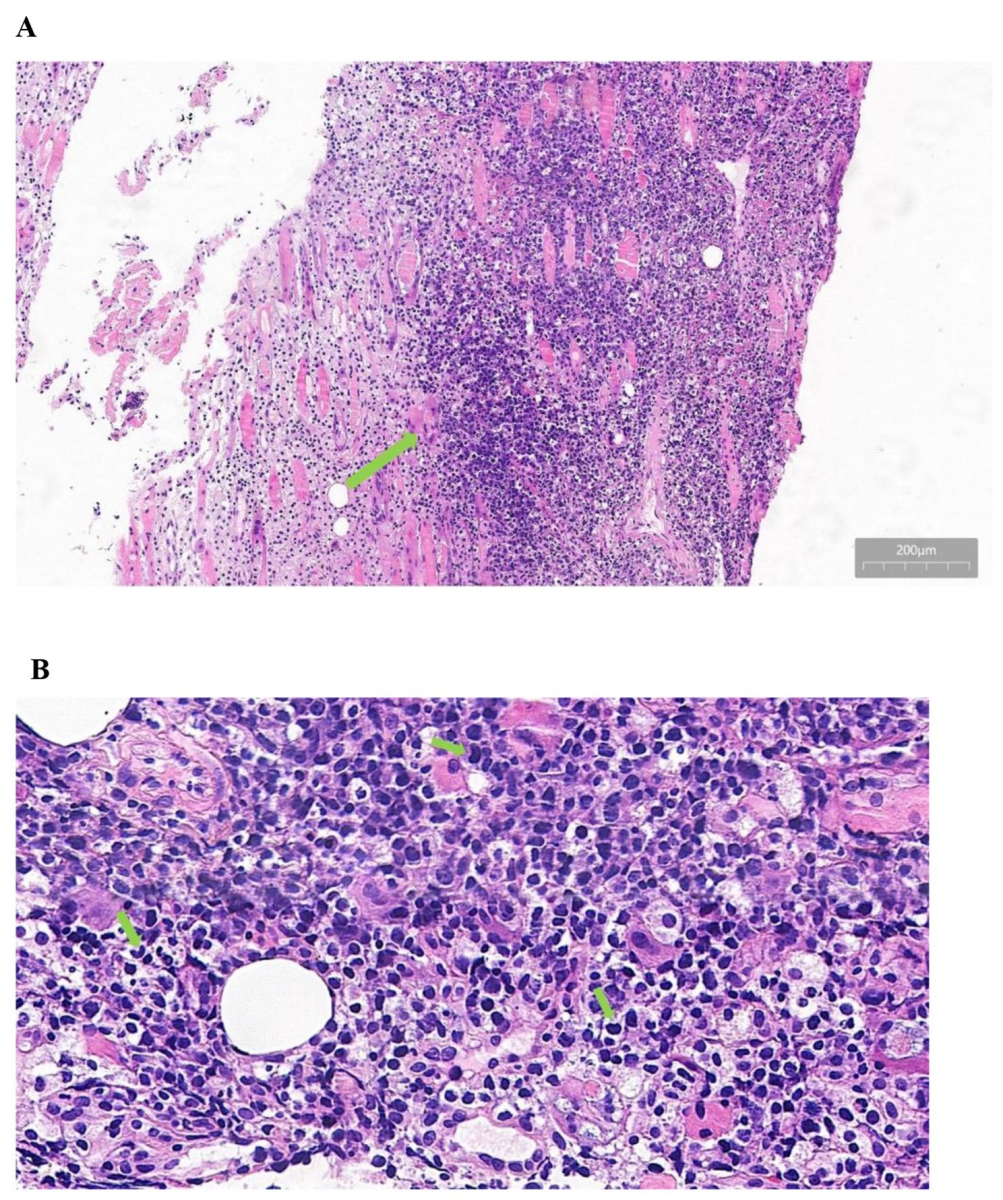
Histopathology a sample of the left temples shows the tumor cells have an anaplastic morphology, with irregularly shaped nuclei, prominent nucleoli, and abundant cytoplasm (**A**-**B**). (Original magnification A 100×, B 400×). Green arrow of Fig. 2B showing the “hallmark” cell with eccentric horseshoe-shaped nuclei.

**Fig. 3:**
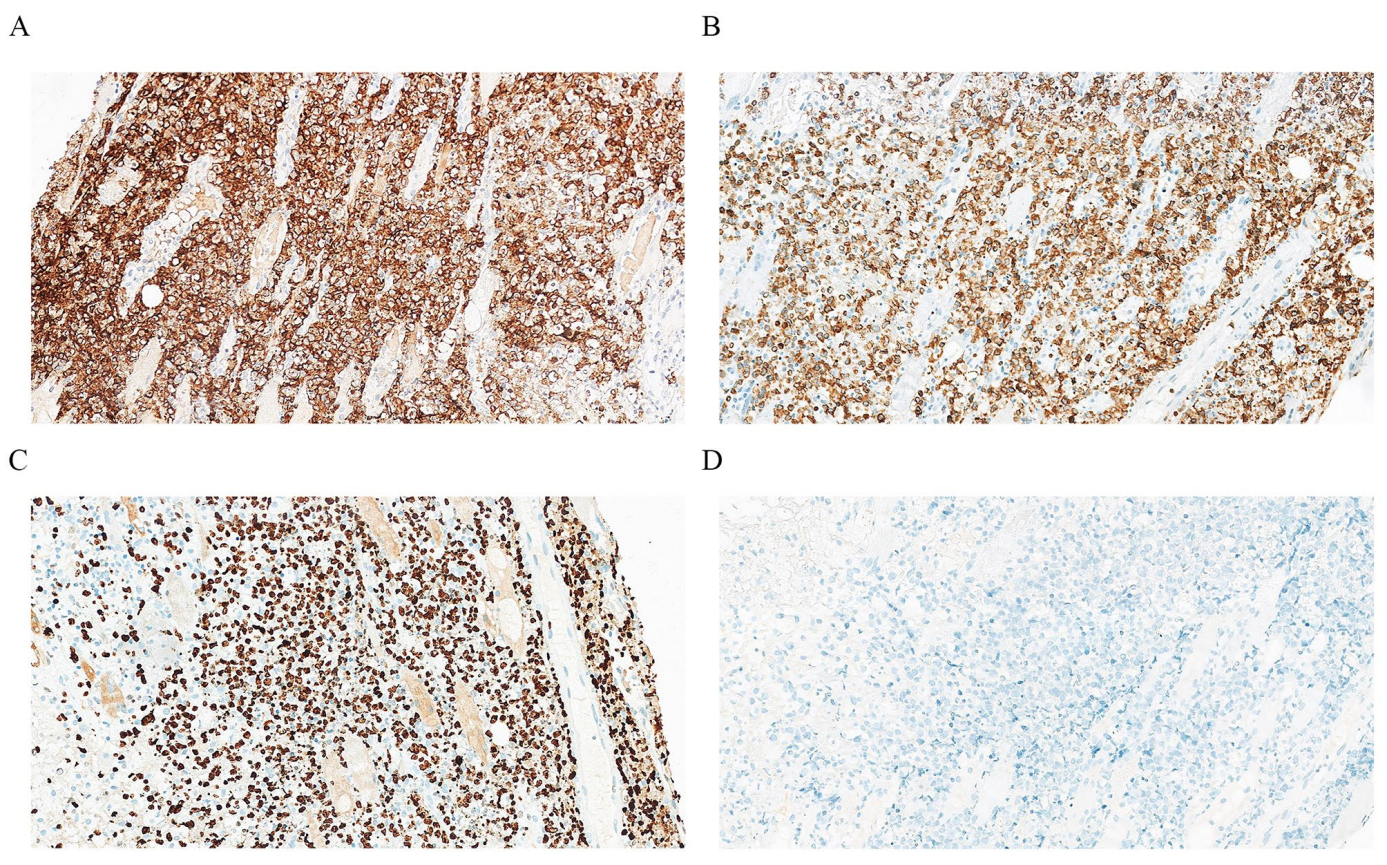
Immunohistochemical analysis showed positivity for (**A**) CD30 protein, (**B**) CD3 protein, and (**C**) Ki67 protein. (Original magnification × 200). Immunohistochemical analysis showed negativity for (**D**) ALK(SP8) protein. (Original magnification × 200)

The definitive histopathological examination showed necrosis and extensive involvement of the left temples by a diffuse infiltrate containing large, atypical cells [Fig. 2A, B]. The immunohistochemical panel showed that the cells were positive for CD30[Fig. 3A], CD3 [Fig. 3B]and Ki67 [Fig. 3C]. They were negative for ALK (SP8) [Fig. 3D], CD5, CD8, CD20 and PAX5. To ensure no metastasis, a positron emission tomography (PET) scan was conducted, and no suspicious metastasis was found.

Considering the findings, the diagnosis of PC-ALCL was made. The patient was admitted to the lymphoma department for systemic chemotherapy with CDOP (cyclophosphamide, vindesine, liposome, dexamethasone, and prednisone), and the patient’s clinical status improved after the first treatment cycle.

## Discussion and conclusions

PC-ALCL is a rare ALK-negative T-cell neoplasm with an annual incidence of 10 cases per million individuals [12]. It is commonly seen in people between 50 and 70, but it has also been reported in pediatric cases [13]. Around 80% of cases present with a localized cutaneous nodule or papule, and aggressive extracutaneous dissemination occurs in only about 10% of cases and it is most likely to involve regional lymph nodes [5, 14, 15]. Histologically, the tumor tissue is comprised of a dense infiltrate of large, irregular lymphoid cells that extend into the dermis or subcutaneous tissue with a variable proportion of “hallmark” cells showing typical horseshoe-shaped nuclei [16]. Immunophenotypically, the tumor cells express strong (> 75%) expression of CD30 and are typically negative for ALK [17]. However, CD30 is not specific for PC-ALCL, and further evaluation is needed in conjunction with other IHC panel, including T‐cells (i.e., CD3, CD5 CD8), B‐cells (CD20, PAX5), Hodgkin lymphoma (PAX5). If there is a strong and uniform expression of CD30 and loss of expression of one or more T-cell antigens PC-ALCL should be considered [18, 19].

Infratemporal space infection is a rare condition that is typically caused by tooth extraction or the spread of infection from other masticatory spaces [20]. The symptoms vary depending on the affected anatomical feature and may include pain, fever, trismus, swelling, and even neurosensory deficit [21]. Carcinoma of facial skin occurring in the temporal region mainly includes SCC and BCC, of which basal cell carcinoma is more common. SCC presents as firm, keratotic papules or red scaly plaques and smooth nodules. BCC usually develops on sun-exposed areas with pearly or translucent papules or nodules [22]. Due to the diverse clinical features of PC-ALCL in the maxillofacial region and its similarity to the aforementioned diseases, the diagnosis of PC-ALCL is challenging.

In this clinical case, the lesion occurring in the temporal region shows diffuse swelling with cumulative regional lymph nodes, combined with the history of diabetes mellitus and misdiagnosis of the first biopsy; all these factors associated with the clinical aspect of the lesion led us to the initially suspected of infratemporal space infection. However, the patient did not refer to a history of tooth extraction or other space infection and was missing the liquefaction foci on imaging findings, showing only enlarged lymph nodes and tissue swelling. These clinical aspects should be contradicted by infratemporal space infection. Due to the rare incidence of PC-ALCL, the identification is very difficult to perform without pathological examination. Clinicians need to make an initial diagnosis of PC-ALCL based on clinical features and integrate complex interdisciplinary information with pathologists. The use of HE staining and a comprehensive IHC panel are essential to diagnose PC-ALCL and prevent misdiagnosis.

## Data Availability

All data generated or analysed during this study are included in this published article.

## Abbreviations

PC-ALCL: Primary cutaneous anaplastic large-cell lymphoma
ALCL: Anaplastic large cell lymphoma
NHL: non-Hodgkin’s lymphoma
CRP: C-reactive protein
HE: Hematoxylin and eosin
IHC: Immunohistochemistry
CBCT: A cone beam computed tomography
PET: positron emission tomography
